# Hemorrhagic stroke—Pathomechanisms of injury and therapeutic options

**DOI:** 10.1111/cns.13225

**Published:** 2019-10-03

**Authors:** Neeraj Chaudhary, Aditya S. Pandey, Xiaoying Wang, Guohua Xi

**Affiliations:** ^1^ Department of Neurosurgery University of Michigan Ann Arbor Michigan; ^2^ Department of Radiology University of Michigan Ann Arbor Michigan; ^3^ Departments of Neurosurgery and Neurology Tulane University New Orleans Louisiana

Each year, approximately 5.5 million people die due to a stroke in the world^1^ and this number could continue to rise with an aging population. The factors leading to a stroke are nonmodifiable or genetic and modifiable such as smoking, hypertension, diabetes mellitus, diet rich in fats and salt, and other environmental factors. The modifiable factors serve as the key in preventing ischemic stroke and hemorrhagic stroke. Our understanding of the pathomechanisms of brain injury in hemorrhagic stroke (axial hemorrhage: intracerebral hemorrhage (ICH), extra‐axial hemorrhage: subarachnoid hemorrhage (SAH), intraventricular hemorrhage (IVH), epidural hemorrhage, and subdural hemorrhage), the less common subtype of stroke, has improved significantly during the past two decades. The bench side research in the field of cellular injury post‐ICH culminated in the first world ICH meeting at University of Michigan in 2005, under the guidance of Dr Julian T. Hoff. In the same vein, the current issue of CNSNT is a collection of various articles depicting the cellular/subcellular level of understanding of acute, subacute, and chronic cell injury in hemorrhagic stroke and some related therapeutic options.

Cerebral hemorrhage leads to direct cerebral injury and then secondary injuries related to edema formation, inflammation, and a rise in intracranial pressure with consequent decline in cerebral perfusion pressure. Early brain injury following an episode of hemorrhage is mediated partly by early erythrolysis, within the first 24 hours.^2^ The hemoglobin burden then gets digested by heme oxygenase, thus releasing the neurotoxin, iron. Iron‐mediated toxicity in the form of tissue edema, inflammation, and cellular death has been demonstrated in the rat and pig ICH models in great detail and has been reproduced by independent laboratories. The release of iron has effects on the blood‐brain barrier, endothelial‐pericyte interaction, veins, and glymphatics which mediate glial and axonal injury. Ischemic preconditioning is being investigated for its role in potential protection from delayed ischemic neurological deficit that results in 20%‐30% of patients with SAH due to vasospasm. In this regard, it is interesting to note that hyperbaric oxygen pretreatment in rat ICH model reduces related injury by regulating polarization of microglial cells.^3^ This shows that there is room to investigate the oxygen equilibrium in the cellular and extracellular environment and how it can be modulated to offer neuroprotection in hemorrhagic stroke.

White matter injury post‐ICH/SAH is an important pathological factor in patient outcomes. White matter injury can be secondary to the mechanical injury post‐hemorrhage, and it can also occur as a combination of injury to oligodendrocytes and damage to axons. Mediators like lipocalin‐2 have been demonstrated to be protective to hemorrhage‐mediated white matter injury in a mouse SAH model.^4^ Unfortunately, white matter injury after ICH has not been well studied.^5^ In the current issue, Kang and Yao review the role of oligodendrocytes in ICH inducing white matter injury.^6^

Spontaneous or secondary IVH is a marker of poor prognosis for hemorrhagic stroke. Severe IVH, caused by extension from ICH or SAH, leads to hydrocephalus and has a greater risk of hemorrhage‐associated morbidity. However, the mechanisms of brain hemorrhage‐induced hydrocephalus are not well understood. In this special issue, Wan et al demonstrated that activation of epiplexus cells is associated with SAH‐ and thrombin‐induced hydrocephalus.^7^ The effect of thrombin in hydrocephalus development may be through protease‐activated receptor‐1.^8^

In parallel, translation of the animal ICH model cellular understanding of pathomechanisms to human subjects is gradually coming to fruition. This is coming in the form of magnetic resonance imaging (MRI) which can pick up susceptibility from iron in the hemoglobin and otherwise in the intracellular and extracellular environment. Investigators have demonstrated that just like in rat and pig ICH models^9^, ^10^, tissue iron in the periphery of the hematoma can be picked up in the humans too^11^. The concentration of iron in the hematoma is very high but currently there is no reliable measurement of iron concentration can be performed within it. On the other hand, in the periphery of the hematoma, such susceptibility compared to the tissue background can be picked up and quantified in the human subjects.^11^ The investigators at University of Michigan have also put forth the hypothesis that whether hemoglobin and its degradation products are within or without red blood cells, it dictates the type of signal on MRI.^12^ This phenomenon of T2* non‐hypointensity, related to early erythrolysis, demonstrated in the animal models, is being investigated in humans with MRI.^13^

Currently, no definite intervention has been shown to be beneficial for ICH, be it hematoma evacuation^14^, ^15^ or iron chelation treatment such as deferoxamine.^16^ However, there is significant enthusiasm as MISTIE III has shown that patients who have greater than 75% of their ICH evacuated are clinically significantly better than those managed conservatively. In addition, iDEF has reported better clinical results at 6 months post‐ICH that approach significance for those patients receiving deferoxamine vs. placebo. In both of these treatments, preventing secondary injury to brain tissue is mediated favorably by the removal of deposited iron. Thus, a well‐validated MRI‐based assessment of tissue iron concentration in the periphery of the hematoma will be very useful for monitoring treatment of hemorrhagic stroke patients with iron chelation agents (eg, deferoxamine). The evaluation of iron concentration post‐ICH/SAH treatment could serve as a marker not only of treatment but also of long‐term clinical outcome.

In summary, the future of hemorrhagic stroke research is looking bright and imaging‐based cellular brain injury pathways can be better established and understood on MRI. A lot more work is needed to validate MRI‐based biomarkers and its correlation with functional outcome in patients with hemorrhagic stroke. The editors commend the authors for presenting a collection of articles at the cutting edge of understanding the underlying mechanisms of cerebral injury post‐ICH and post‐SAH.

## CONFLICT OF INTEREST

We declare that we have no conflict of interest.
